# Antioxidants in Longevity and Medicine 2014

**DOI:** 10.1155/2015/739417

**Published:** 2015-05-11

**Authors:** Narasimham L. Parinandi, Nilanjana Maulik, Mahesh Thirunavukkarasu, David W. McFadden

**Affiliations:** ^1^Division of Pulmonary, Allergy, Critical Care and Sleep Medicine, Department of Medicine, Dorothy M. Davis Heart and Lung Research Institute, The Ohio State University Wexner Medical Center, 473 W. 12th Avenue, Room 601, Columbus, OH 43210, USA; ^2^Department of Surgery, University of Connecticut Health Center, 263 Farmington Avenue, Farmington, CT 06032, USA

The reactive oxygen species (ROS) and reactive nitrogen species (RNS) generated from the enzymatic and nonenzymatic sources from superoxide (O_2_^∙−^) and nitric oxide (NO), respectively, are established to cause oxidative, nitroxidative, nitrative, and nitrosative stresses in the biological systems. Scientific evidence is mounting in favor of the involvement of ROS-induced oxidative stress and RNS stress in diverse pathophysiological states in experimental animal models and humans including the cardiovascular, cerebrovascular, and renal diseases to name a few. Among many proposed and tested theories of aging, the oxidative stress theory of aging underscores the ROS-induced damage as being instrumental in the progressive and unalterable alterations at the molecular, cellular, and organ levels during aging and shortening of life span. The balance between the ROS/RNS and antioxidants is highly critical to maintain the cellular health and any alteration in that balance caused by either the elevation of production of ROS/RNS or decrease of antioxidant status in the cells will lead to diverse pathophysiological states. Antioxidants (enzymatic and nonenzymatic), either endogenous or exogenous, are known to counteract the deleterious actions of ROS/RNS leading to protection against the oxidative stress and RNS stress. Therefore, this special issue focuses on the possibilities of protection against the ROS-induced oxidative stress and RNS stress which could potentially impact aging, life span, and diseases among humans. Several experts in the fields of ROS and RNS biology have contributed original research and review articles highlighting the state-of-the-art cellular and molecular mechanisms and protection of pathophysiology of oxidative stress and RNS stress in diseases, aging, and age-associated pathophysiological states.

The articles published in this special issue are broadly grouped into (1) conditions that elevate oxidative stress, (2) conditions that decrease/attenuate oxidative stress, and (3) aging and oxidative stress.

*Articles on Conditions/Factors That Elevate Oxidative Stress*. As the deficiency of estrogen in postmenopausal women is shown to have a direct impact on their susceptibility to cardiovascular diseases, A. Pósa et al. conducted a study on the endogenous estrogen-mediated heme oxygenase (HO) regulation in rats under conditions of menopause. In this study, the investigators used an estrogen depletion model and showed that the sensitivity of the animal model to myocardial ischemia was associated with the suppression of HO activity and expression of HO-1/HO-2 as well as an increase in the secretion of proinflammatory cytokines and biomarkers. Also, the authors demonstrated elevated myeloperoxidase activity as well as the depression of the electrocardiogram ST segment in the estrogen-depleted rats which was exacerbated by inhibiting heme oxygenase activity. Obstructive sleep apnea (OSA) is identified as an independent risk factor in cardiovascular diseases through a state of intermittent hypoxia (IH) that induces oxidative stress and inflammation. The endogenous transition metal-binding protein, metallothionein (MT), is identified as an inducible antioxidant that may offer protection against oxidant-induced damage. Along these lines, S. Zhou et al. conducted the study on the deletion of MT which exacerbated the IH-induced oxidative and inflammatory injury in the aorta. The results of this study suggested that IH could be an important condition/factor that could lead to aortic injury through oxidative stress and inflammation, wherein MT might protect against the IH-induced vascular injury. Goeckerman therapy (GT, skin treatment with a combination of 3% crude coal tar ointment and UV irradiation) is a current form of skin treatment for children suffering from plaque psoriasis, a multifactorial skin disease. L. Borska et al. carried out a prospective cohort study on children with plaque psoriasis receiving the GT and showed adverse (side) effects of the treatment on the subjects following the GT. The authors reported an increase in several oxidative stress markers including the oxidative nucleic acid damage, formation of the benzo(a)pyrene-7,8-diol-9,10-epoxide-DNA (BPDE-DNA) adducts, and chromosomal abnormalities (index of genotoxicity) in lymphocytes of the subjects following the GT. However, the authors acknowledged that elevated levels of oxidative stress and genotoxic biomarkers might not necessarily be associated with the side effects of the GT and additionally the treatment showed significant clinical benefit. L. A. Rabelo et al. provided a comprehensive review on arginase as a critical prooxidant mediator in the binomial endothelial dysfunction atherosclerosis. This review focused on the involvement of arginase in vascular endothelial function and its role in atherosclerosis. Also, the authors discussed adverse actions of arginase in relation to oxidative stress and in various metabolic diseases. The authors specifically described the role of arginase in endothelial function and the production of atherosclerotic lesions with an emphasis on the mechanisms of regulation of the enzyme and potential development of strategies of pharmacological intervention of CVDs.

*Articles on Conditions/Factors That Decrease Oxidative Stress*. Currently, there is a dearth of evidence/information on the interaction(s) between biomarkers of oxidative stress and chemokines in humans, specifically among adults without overt clinical disease(s). Therefore, Y. Li et al. focused on studying the relationship between antioxidants and markers of lipid peroxidation along with different chemokines in adults. In this study, the authors observed a positive correlation between total antioxidant status and different chemokines. However, the authors were unable to establish association between chemokines and biomarkers of lipid peroxidation. Mitochondrial respiratory chain dysfunction and elevated oxidative stress are markedly encountered in the brain during aging. With the aim to investigate whether natural products would alleviate/mitigate the age-associated cerebral mitochondrial respiratory dysfunction and enhanced oxidative stress, A. B. de Sá-Nakanishi et al. investigated the effects of treating old rats with aqueous extracts of* Agaricus blazei* (medicinal mushroom) on oxidative and functional outcomes of the brain tissue and brain mitochondria of 21-month-old rats. The results of this study revealed that the intragastric administration of mushroom extract offered protection against elevated oxidative stress (ROS production and lipid peroxidation) and increased the antioxidant (nonenzymatic and enzymatic) status in the brain of old rats. Furthermore, administration of the mushroom extract showed enhancement of the activity of different mitochondrial respiratory enzymes and mitochondria-coupled respiration. This study revealed that natural products present in the medicinal mushroom extract offered protection against the age-associated oxidative stress and enhanced mitochondrial activity in the brain of old rats. The natural product, 2,3,5,4′-tetrahydroxystilbene-2-O-*β*-D-glucoside (TSG) obtained from the Chinese herb,* Polygonum multiflorum* (traditionally used as an antiaging plant remedy), was investigated by C. Büchter et al. for its actions to increase the life span and stress resistance of* Caenorhabditis elegans*. This study revealed that TSG increased the antioxidative potential and stress resistance and prolonged life span in the nematode as compared to the actions exhibited by resveratrol. In a review article, S. Zhou et al. extensively discussed the role of nuclear factor-erythroid (NF-E) 2-related factor 2 (Nrf2) in cardiac remodeling and heart failure. In this review the authors described the role of Nrf2 in various antioxidative phenomena, cytoprotection, inducible expression of antioxidant genes, and phase II detoxifying enzymes in the cardiovascular system. Their focus of this review was to disclose the antioxidative capability of Nrf2 as well as its protective actions against cardiac remodeling and heart failure. S. C. Khor et al., in their review, discussed in detail vitamin E in sarcopenia with the current evidences on its role in prevention and treatment of sarcopenia. This review revealed vitamin E as a potential treatment for sarcopenia, a disease of the old age associated with decreased muscle mass and strength. In addition, the authors discussed the protective/beneficial actions of vitamin E in combating oxidative stress and improving muscle health. Moreover, the authors expressed that further studies are warranted to establish the specific mechanism(s) of protective actions of vitamin E in sarcopenia.

*Articles on Aging and Oxidative Stress*. Currently, evidences are accumulating in support of micro-RNAs (miRNAs) as the posttranscriptional regulators of aging. Aiming at this, S. G. S. Khee et al. studied the expression of senescence-associated miRNAs and their target genes in cellular aging and their modulation by tocotrienol-rich fraction (an isomer of vitamin E) in young and old human diploid fibroblasts. This study suggested that tocotrienol prevented cellular senescence of human diploid fibroblasts through the modulation of certain senescence-associated miRNAs and the expression of target genes. Sirtuins (silent information regulator proteins) are focused to prevent oxidative damage that threatens female fertility. SIRT1 and SIRT3, the main members of the sirtuin family, have been recognized as the sensors and guardians of redox status in oocytes, granulosa cells, and embryos. Taking this into consideration, C. Tatone et al. reviewed in detail the functions of sirtuins in female fertility and their possible role in oxidative stress and aging. This review discussed the role of sirtuins in the prevention of female infertility arising from oxidative stress. More specifically, the authors addressed the actions of SIRT1 and SIRT3 and their functions as sensors and protectors of oxidative stress at various stages of the reproductive cycle. The authors envisioned future studies that would lead to the use of sirtuins as targets to rectify female infertility. L. Marseglia et al. discussed in detail in their review the oxidative stress-mediated aging during the fetal (pregnancy) and perinatal stages focusing on the onset of common disorders in the newborn as an outcome of the early aging process. The authors proposed the onset of aging prior to birth. This review described the significant role of oxidative stress in the pathogenesis of various pregnancy-related and neonatal disorders.

Attenuating or inhibiting the overwhelming production and deleterious actions of ROS and RNS by treatments with several antioxidants is increasingly becoming sought-after therapeutic strategies to treat age-related diseases and extend the life span wherein the ROS-induced oxidative stress and RNS stress play crucial roles. The goal of this special issue is to disclose the latest understanding of the mechanisms and protection of oxidative stress and RNS stress and to create opportunities to move the field forward that will impact the oxidative stress-mediated diseases and aging in humans. Hence, the articles in this special issue contributed by the experts in the fields of oxidative and RNS stress biology will nurture continued discoveries towards better understanding of the mechanisms and protection of oxidant injury, oxidative stress-mediated diseases, and age-related pathophysiological states.

*Narasimham L. Parinandi* *Narasimham L. Parinandi* *Nilanjana Maulik* *Nilanjana Maulik* *Mahesh Thirunavukkarasu* *Mahesh Thirunavukkarasu* *David W. McFadden* *David W. McFadden*

